# Protective ventilation in ARDS: as soon as possible. An immediate use of HFOV

**DOI:** 10.1186/1757-1626-1-124

**Published:** 2008-08-22

**Authors:** Philippe Ph Goutorbe, Yves Y Asencio, Julien J Bordes, Ambroise A Montcriol, Bertrand B Prunet, Eric E Meaudre

**Affiliations:** 1 Military teaching hospital Sainte Anne ICU department, Toulon, France

## Abstract

**Objective:**

To report the immediate use of High-Frequency Oscillatory ventilation in an adult acute respiratory distress syndrome.

**Design:**

Case report.

**Setting:**

Intensive care unit at the Military Teaching Hospital of Toulon.

**Patient:**

A 64-yr-old Caucasian male who developed acute respiratory distress syndrome in the course of severe falciparum malaria.

**Intervention:**

Initial use of HFO to minimise ventilator-induced lung injury.

**Measurement and Main Results:**

Rapid improvement of PaO2/fraction of inspired oxygen from 172 mmHg (NIV) to 310 mmHg with HFO. No ventilator-induced injury on CT scan after 5 days of invasive ventilation.

**Conclusion:**

In contrast with previous studies, we successfully used lung protective ventilation with HFO immediately. Further studies, with immediate, rather than rescue use of HFO ventilation, are needed.

## Introduction

Although mechanical ventilation is often lifesaving for patients with acute respiratory distress syndrome (ARDS), it can itself cause further lung injury: ventilator induced lung injury (VILI)[1]. Three mechanisms lead to lung injury: gross air leaks (barotrauma), overdistension (volutrauma) and the cyclic opening and closing of unstable lung units (atelectrauma). This results in structural damage and biochemical injury with the systemic inflammatory response syndrome.

Lung-protective ventilation strategies have been developed to minimise VILI. Amato et al described in 1998 improved outcome in ARDS patients ventilated with small tidal volumes and positive end-expiratory pressures (PEEP) slightly above the lower inflection point on the pressure volume curve[2]. The ARDS net investigators reported in 2000 a 9% decrease in absolute mortality of patients with ARDS using small tidal volumes with a mean PEEP of 10 cmH2O[3].

High-frequency oscillatory ventilation (HFOV) can provide adequate gas exchange with very small tidal volumes and thus achieve protective lung ventilation[4]. We report a case of malaria-related ARDS in which HFOV was used from the onset of ventilation. The early use of this protective ventilation may explain the absence of structural lung damage on the CT scan after 5 days.

## Case report

The patient was a 64-yr-old caucasian with a history of fever following a stay in Ghana. He had not taken any anti-malarial chemoprophylaxis. On admission he was confused, thrombopenic (36000 giga per ml), hyperbilirubinemic (77 micromoles/l) and in mild acute renal failure (urea 16.9; creatinine 130 micromoles/l). Blood examination showed a high concentration of plasmodium falciparum (parasitemia = 30%).

**Figure 1:**
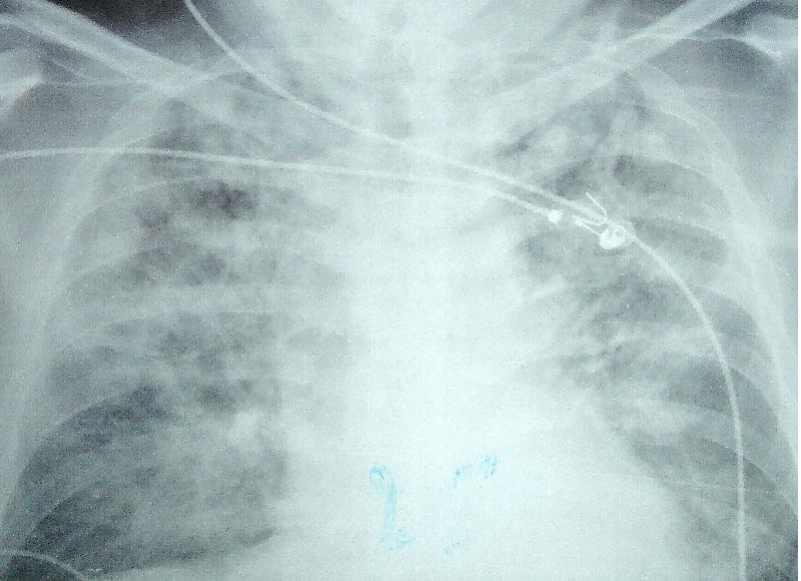
Chest X ray just before intubation.

Treatment included intravenous infusion of quinine and dalacine. On day 6 he presented with acute respiratory failure. The chest X ray showed diffuse pulmonary oedema (fig 1). Echocardiography showed a normal ejection fraction and good diastolic function (E/Ea = 5) with a low brain natriuretic peptide (99 micromoles per ml), which thus eliminated any cardiac cause for the edema. Fiber-optic guided distal protected lavage showed no bacterial culture. We concluded that the patient had an extrapulmonary acute respiratory distress syndrome triggered by malaria.

Non-invasive ventilation was tried for two hours without improvement in gas exchange (PaO2/FiO2 = 170 mmHg with FiO2 of 0.5). The patient was intubated and HFOV started immediately, using a 3100B high-frequency oscillatory ventilator (SensorMedics, Yorba Linda, CA).

Initial HFO settings was:

Bias flow 40 L/min.

FiO2 0,7.

Inspiratory time = 32%.

MPaw = 27 cmH_2_O.

Amplitude (ΔP) = 90 cmH_2_O.

Frequency = 4,5 Hz.

Gas exchange improved rapidly. After four hours, PaO2/FiO2 was 310 mmHg with an FiO2 of 0,7. FiO2 was reduced to 0,4 and Mpaw to 22 cmH_2_O. After 48 hours we returned to conventional ventilation.

**Figure 2:**
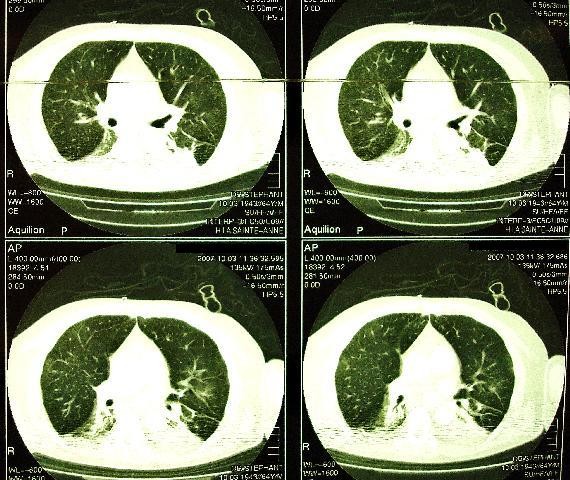
CT Scan on day 5.

After 5 days of invasive ventilation a CT scan, performed during an inspiratory pause of 15 cmH_2_O, showed a structurally normal lung, without any ventilator induced lung injury (Fig 2). The patient was successfully extubated after 11 days of invasive ventilation, and was discharged from ICU on day 19. He returned home on day 25 without any sequellae.

## Discussion

Adequate ventilation is an important endpoint in the management of in ARDS. Several protective ventilation strategies have been described, of which small tidal volume with a mean PEEP of 10 cmH_2_O is the most common[3]. Extra corporeal gas exchange is also used with very small tidal volumes and low frequency. HFOV provides alveolar ventilation with very small tidal volumes and thus theoretically provide an optimal lung-protective ventilatory strategy[5].

Gas exchange during HFOV depends on different mechanisms: interregional gas mixing between units with different time constants (Pendelluft), convective transport attributable to asymmetry between inspiratory and expiratory profiles, cardiac oscillation, collateral ventilation and Taylor dispersion. These mechanisms allow adequate oxygenation and CO2 clearance with tidal volumes of only 1–3 ml/Kg[6,7]. In addition, the raised mean airway pressure can achieve lung recruitment despite unequal time constants, and prevents end-expiratory alveolar collapse[8].

Treggiari M et al described two types of structural lung damage related to ventilation; air cyst and bronchiectasis[9]. There was a positive correlation between the duration of mechanical ventilation and the amount of lung damage. Moreover a negative correlation was noted between the volume of normal parenchyma and the mean end-inspiratory pressure. In our patient the normal CT scan on day 5 was attributed to the immediate protective effects of HFOV.

The immediate use of a lung-protective strategy is justified on theoretical grounds. However, the time at which HFOV should be initiated is controversial[10]. Four trials, of which three were prospective, identified the duration of prior conventional ventilation as an independent predictor of mortality in ARDS. A systematic review of nine studies found that the duration of conventional ventilation prior to starting HFOV was significantly greater in non-survivors. When adjusted for age and APACHE II score, each extra day on conventional ventilation was associated with a 20% increase in mortality, although this association disappeared when baseline pH was included in the multivariate analysis (enable data for five study)[11].

We think that HFOV is a highly lung-protective strategy which should be used in ARDS as soon as possible, especially in early and diffuse ARDS. Further trials with immediate use of HFOV are required to confirm this.

## Abbreviations

ARDS: Acute respiratory distress syndrome; HFOV: High-frequency oscillatory ventilation; Mpaw: Mean airway pressure; PEEP: Positive end-expiratory pressures; VILI: Ventilator induced lung injury.

## Competing interests

The authors declare that they have no competing interests.

## Authors' contributions

All the authors contribute to the treatment of this patient.

##  Consent

Written informed consent was obtained from the patient for publication of this case report and accompanying images. A copy of the written consent is available for review by the Editor-in-Chief of this journal.
